# A stinging case of misinterpretation

**DOI:** 10.1016/j.jacig.2026.100656

**Published:** 2026-02-09

**Authors:** Carmen Vidal, Margarita Armisén, Xesús Feás

**Affiliations:** a Allergy Service, Complejo Hospitalario Universitario de Santiago, Santiago de Compostela, Spain; b Academy of Veterinary Sciences of Galicia, Edificio Escola Galega de Administracion Publica, Santiago de Compostela, Spain

To the Editor:

Giangrande et al report a case series of 6 patients who experienced systemic reactions after stings by *Vespa velutina nigrithorax* (VVN) despite receiving *Vespula* spp venom immunotherapy at 4 different hospitals in Spain.^1^ This is an important and timely issue because the expansion of VVN across Europe has resulted in a growing number of sensitized patients, and the recent availability of VVN-specific extracts has opened new opportunities for diagnosis and treatment. Nevertheless, some of the conclusions expressed in the article by Giangrande et al^1^ require clarification.

First, 4 of the 6 patients had previously experienced anaphylaxis not in response to VVN but rather in response to *Vespula* spp and therefore received immunotherapy with the latter venom according to current clinical standards.^2^ It is not methodologically appropriate to consider such a response “treatment failure” because these patients reacted to a sting from a different insect species during or after therapy. Such reasoning leads to a misleading interpretation: suggesting that all patients with *Vespula*-induced anaphylaxis should also receive VVN immunotherapy does not seem justified unless there is proven sensitization to this species with clinical implications.

Second, the 2 patients who experienced anaphylaxis after VVN stings and were treated with *Vespula* spp venom could indeed be considered cases of therapeutic failure. However, it must be acknowledged that the efficacy of Hymenoptera venom immunotherapy, even with the culprit insect venom, is not 100%.^2^ Therefore, occasional failures are not unexpected, which weakens the validity of drawing broad conclusions from so small a sample.

In addition, Giangrande et al^1^ use the term *frequency* without providing a denominator, which would allow proportions to be calculated. With only 6 cases across 4 hospitals and without information regarding the total number of patients treated in each center, estimating prevalence or incidence of treatment failure is not possible. In our own experience, which has involved more than 300 patients who experienced anaphylaxis in response to VVN and were treated with *Vespula* immunotherapy, clinical tolerance of stings in the field has been the rule.^3^^,^^4^ Moreover, among the more than 15 cases of controlled repeat stings, only 1 failure was observed, and that patient is now successfully receiving VVN-specific immunotherapy.^4^

From an immunologic perspective, interpretation of the reported cases also deserves caution. Some patients may have had double sensitization (to *Vespula* and VVN), and *Vespula* extracts do not adequately cover all relevant VVN allergens, such as dipeptidyl peptidase IV, that have recently been identified in VVN venom.^5^ It may account for isolated failures without implying a general lack of efficacy of *Vespula* immunotherapy.

In conclusion, although this case series provides interesting observations and emphasizes the need for comparative studies between *Vespula* and VVN immunotherapy, interpretation of the data should be more cautious. Presenting 6 isolated cases as evidence of a high frequency of treatment failure is premature and may mislead clinical practice. The availability of molecular diagnosis and VVN-specific extracts will, in the near future, help to define more clearly which patients will benefit most from each therapeutic approach.

*Editor’s note: There is no accompanying reply to this correspondence.*
